# Influence of Synthesis Route on Composition and Main Properties of Mullite Ceramics Based on Waste

**DOI:** 10.3390/ma18051098

**Published:** 2025-02-28

**Authors:** Alina-Ioana Badanoiu, Stefania-Paula Stoleriu, Alexandru-Cosmin Carocea, Mihai-Alexandru Eftimie, Roxana Trusca

**Affiliations:** Science and Engineering of Oxide Materials and Nanomaterials Department, Faculty of Chemical Engineering and Biotechnology, National University of Science and Technology Politehnica Bucharest, 1-7 Gh. Polizu Street, Sector 1, RO-011061 Bucharest, Romania; alina.badanoiu@upb.ro (A.-I.B.); caroceacosmin@gmail.com (A.-C.C.); mihai.eftimie@upb.ro (M.-A.E.); roxana.trusca@upb.ro (R.T.)

**Keywords:** mullite, waste, solid-state reaction, precipitation, composition, microstructure, properties

## Abstract

Mullite, 3Al_2_O_3_·2SiO_2_, is a material with excellent thermal and mechanical properties. Two types of waste sand, rich in impurities, were employed as precursors for mullite ceramic synthesis. Two different synthesis routes were used: (i) solid-state reactions involving a sand and bauxite mixture, and (ii) precipitation synthesis, where alumina was deposited on sand particle surfaces; the sintering process was performed at temperatures ranging from 1300 °C to 1400 °C. Mullite was obtained as the main phase when the ceramics were obtained by solid-state reactions opposite to the second method, in which a composite ceramic with a specific microstructure, i.e., sand particles embedded in a matrix formed by alumina crystals, was assessed by electronic microscopy. The main properties, i.e., the apparent density, open porosity, compressive strength and thermal expansion coefficient (CTE) of the obtained materials were influenced by the composition and microstructure as well as the sintering temperature. The ceramics in which mullite was the main phase had slightly lower CTE’s and did not exhibit any phase transition in the 20–900 °C range. The results presented in this article highlight the importance of the synthesis route correlated with the nature of the precursors, the type and amount of impurities and the sintering temperature.

## 1. Introduction

Mullite, with the chemical formula 3Al_2_O_3_·2SiO_2_ (or A_3_S_2_), has remarkable thermal and mechanical properties [1,2,3,4]. Mullite has a high melting point (1840 °C); thus, mullite ceramics present good mechanical strengths at high temperatures, have low thermal conductivity and have good resistance to creep and thermal shock [2,3]. Moreover, these materials have also good chemical resistance; thus, mullite ceramics are nowadays used as refractory materials, protective coatings, heat shields, crucibles, thermocouple tubes, components for gas turbine engines, etc. [1,2,4,5].

Mullite can be obtained from a wide range of precursors using various synthesis methods. The conventional way to obtain mullite is the solid-state reaction of silica, alumina or clay minerals such as kaolin, sillimanite, kyanite, pyrophyllite or andalusite [4,6,7]. Mullite monolith ceramics are mainly produced by solid-state reactions and are called sintered mullite. The synthesis temperature for sintered mullite ranges between 1600–1800 °C, depending on the alumina content, fineness and homogeneity of the raw mix [4]. Fused mullite can be obtained by melting fused silica, quartz sands and alumina at temperatures higher than 2000 °C, casting the mixture into molds and cooling at room temperature. This type of mullite ceramic has a lower content of impurities and, thus, a better microstructure [4], but the high thermal treatment temperature makes this process very expensive. Other methods used to obtain mullite coatings, films or powders include the sol–gel [8,9,10], chemical vapor deposition (CVD) [11,12] or spray pyrolysis methods [13,14].

Given the new EU regulations regarding the circular economy [15], the valorization of various types of waste, including mineral ones, in value-added products represent a topical subject [16,17,18]. In this context, and due to the high cost of alumina or silica powders, as well as due to the increasing reduced availability of high-alumina-containing clays, an important amount of research is focused on the use of alternative raw materials (wastes) for the synthesis of sintered mullite ceramics [1,2]. Rice husk ash (waste from the combustion of rice husk) [19], kaolin waste produced during mining process [20], ceramic wastes from the manufacture of ceramic tiles [21], photovoltaic silicon waste [22], fly ash from coal-fired power plants [3,23,24] or aluminum sludge [25] have been used, among others, as alternative raw materials to produce sintered mullite ceramics.

Wang et al. [26] obtained mullite ceramic using drift sand and commercially available Al_2_O_3_ powder. The mullite formation temperature was smaller as compared with a reference sample prepared using analytical reagents due to the presence of impurities; other properties, such as the density and mechanical properties, were also improved.

Tripati and Banerjee [27] developed mullite ceramics by the sintering of beach sand containing sillimanite and calcined alumina; they reported that the Al_2_O_3_/SiO_2_ ratio of these mixtures influences the densification, flexural strength and microstructural development of the ceramics. Additions like TiO_2_ [27] or Fe_2_O_3_ [28] favor the densification of these ceramics due to liquid phase formation but can affect the high-temperature flexural strength.

Sánchez-Soto et al. [29] emphasize the important role of vitrification in the mullitization reaction. The presence of impurities in the raw materials could contribute to the increase in the amount of the liquid phase, which can form a glassy phase after cooling, thus enhancing the densification of mullite materials obtained by sintering at 1500–1600 °C.

Fly ash has also been extensively researched as a precursor for the synthesis of mullite ceramics, both dense and porous [2,3,24,30]. To obtain porous mullite ceramics, Dong et al. [3] used a heterogenous precipitation of aluminum hydroxide on the surface of glassy fly ash particles to ensure a homogenous mix of the precursors. A subsequent dry-pressing–sintering process was used to obtain porous mullite ceramics. The alumina coating of fly ash particles prevented the deformation and bulging of the specimens due to the inhibition of sintering shrinkage and enabled obtaining materials with a high open porosity (40–50%) and low relative density (60–90%).

The main objective of this research was to valorize two types of waste sands for the synthesis of mullite ceramics. These sands contained, besides silica, other oxides such iron oxide, alumina or calcium oxide, which can modify the sintering temperature and improve the mullitization process. Two types of precursors were used to bring alumina into the system, i.e., bauxite and aluminum hydroxide, which were obtained by the reaction of an aluminum chloride solution with ammonia solution. This approach supports sustainable manufacturing practices by turning waste into valuable resources.

## 2. Materials and Methods

### 2.1. Materials

Two wastes with a high silica content and bauxite as an alumina source were used for the synthesis of the ceramics. The oxide compositions of these materials are presented in Table 1.

- Waste S is a sand used in the grinding process of foundry products; this explains the high amount of iron oxide compared with waste N;
- Waste N represents sand waste (containing less than 98% SiO_2_ and other impurities) obtained from the processing of natural sand, which is carried out to obtain high-purity silica sand destined for the production of ceramic products;
- Natural bauxite (B) was used as an alumina source.

As can be seen from the data presented in Table 1, waste S contained a higher amount of Fe_2_O_3_ as compared with waste N, which was due to the provenience of this waste, i.e., from the metallurgy industry. On the opposite side, waste N had a higher amount of CaO and alkalis.

The bauxite contained, besides Al_2_O_3_, a high amount of Fe_2_O_3_, silica and other oxides in much lower quantities.

**Figure 1:**
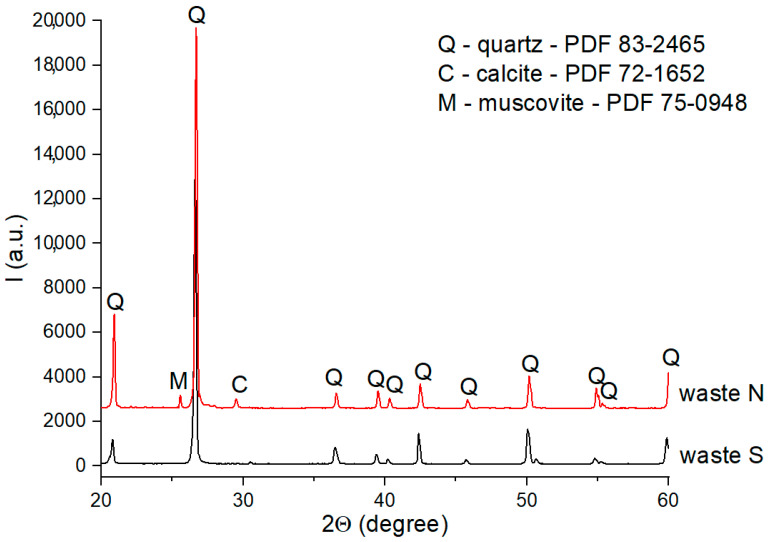
XRD patterns of S and N wastes.

**Figure 2:**
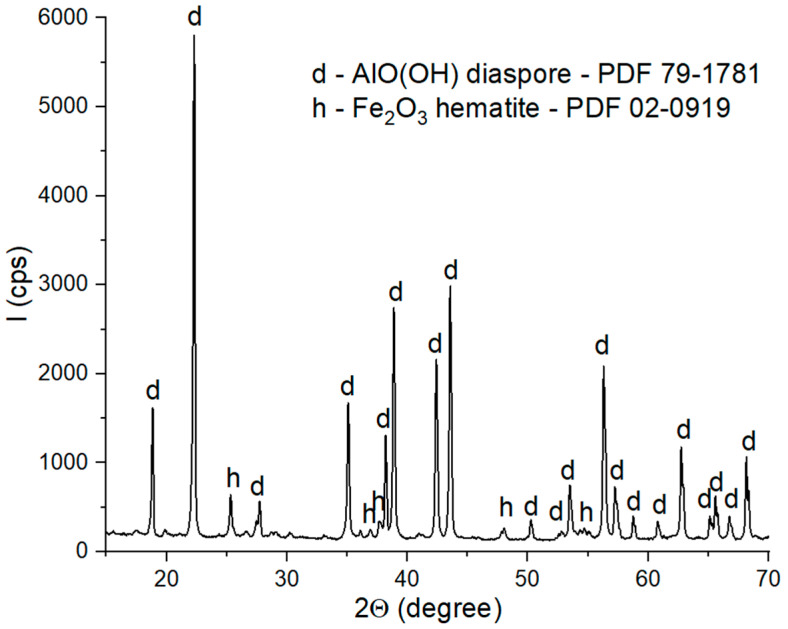
XRD patterns of bauxite.

The XRD patterns of these materials are presented in Figure 1 and Figure 2.

From the XRD patterns of waste S (Figure 1), one can observe the presence quartz (PDF 83-2465) with a high crystallinity degree. Waste N also contained SiO_2_ (quartz) as its main phase; calcite (PDF 72-1652) and muscovite (PDF 75-0948) were also detected in the XRD pattern as secondary phases.

The XRD patterns of the bauxite (Figure 2) showed the presence of two phases: diaspore (AlO(OH)-PDF 79-1781) as the main phase and hematite (Fe_2_O_3_—PDF 02-0910) as the secondary phase.

### 2.2. Ceramics Synthesis

The following two different synthesis routes were used: (i) solid-state reactions involving a sand and bauxite mixture (NB and SB compositions), and (ii) precipitation synthesis, where alumina was deposited on sand particle surfaces (NA and SA compositions); the sintering process was performed at temperatures ranging from 1300 °C to 1400 °C.

For the NB and SB compositions, wastes N or S and bauxite (B) were dosed based on their silica and alumina contents to obtain mullite and then milled (using a planetary ball-mill—Fritsch Pulverisette planetary ball-mill, Idar-Oberstein, Germany) until total pass through a 100 μm mesh sieve, weighed, homogenized and shaped. The mixing was carried out using isopropyl alcohol (using same planetary ball-mill) for better homogenization, thus promoting the precursors’ reaction from the early stages of the sintering process. After homogenization, the powders were dried in an oven (at 60 °C for 24 h) and then uniaxially pressed at 150 MPa into cylinders (with diameters of 13 mm and heights of 12 mm).

A different procedure was used for the NA and SA compositions. The N and S particles were coated with Al_2_O_3_ on the assumption that this procedure would increase the rate of the chemical reaction between the silica and alumina during the thermal treatment. Thus, the S and N wastes were mixed with an adequate volume of aqueous solutions of AlCl_3_ (1 mol⸱L^−1^ aluminum chloride solution obtained from aluminum chloride hexahydrate, chemically pure, Sigma Aldrich, Saint Luis, MI, USA) in order to obtain an alumina–silica molar ratio of 3:2. To avoid waste sedimentation, DARVAN C (high-molecular-weight ammonium polymethacrylate solution, R.T. Vanderbilt Co., Norwalk, CT, USA) was added as a dispersing agent. For aluminum hydroxide precipitation, a 25% ammonia solution (Sigma Aldrich, Saint Luis, MI, USA) was added to these suspensions until the pH reached 12.

The obtained particles covered by precipitate were filtrated and washed until pH = 7. The filtrate was dried at 80 °C for 24 h in an oven, calcined at 350 °C [31] and shaped by uniaxial pressing at 150 MPa into cylinders (with diameters of 13 mm and heights of 12 mm). The ceramics obtained by this method were denominated as NA and SA.

For both synthesis routes, the thermal treatment was performed in an electric kiln (heating rate 10 °C/min) at three different temperatures, i.e., 1300 °C, 1350 °C and 1400 °C, with a plateau of two hours followed by rapid cooling (20 °C/min) to normal temperature. The selected sintering temperatures were lower as compared with those currently used for the sintering of mullite ceramics [4] and were chosen considering the sustainability goal, i.e., to reduce the carbon footprint of the obtained ceramics.

### 2.3. Methods

The mineralogical compositions of the wastes (used as precursors) and obtained ceramics were assessed by X-ray diffraction (XRD). The XRD was performed on a Shimadzu XRD 6000 diffractometer (Shimadzu, Kyoto, Japan). The XRD patterns were obtained using monochromatic CuKα radiation, and the scanning was performed in the range of 2θ = 15−70 degrees.

The microstructure and elemental composition of selected areas were assessed by scanning electron microscopy (SEM), backscattered electron imaging (BSE) and energy-dispersive X-ray analysis (EDX). These analyses were performed on a FEI Inspect F scanning electron microscope (FEI, Hillsboro, OR, USA) equipped with an energy-dispersive X-ray spectrometer. The specimens were covered with a thin conductive coating of gold.

The ceramic properties (apparent density—ρ_a_, and open porosity—P_d_) were determined using the Arthur method [32] and calculated using the following formulae:

ρ_a_ = (m_i_·ρ_x_).m_xa_ − m_x_ (1)

P_d_ = (m_xa_ − m_i_)·100/(m_xa_ − m_x_) (2)

where m_i_ is the initial mass of the sample (g); m_xa_ is the mass weighed in air of the sample saturated with xylene (g); m_x_ is the mass weighed in xylene of the sample saturated with xylene (g); ρ_a_ is the apparent density (g/cm^3^); P_d_ is the open porosity (%); and ρ_x_ is the density of xylene (0.86 g/cm^3^).

The compressive strengths were assessed for the ceramic cylindrical samples sintered at different temperatures using a LFM 50 kN no. 596 testing machine (Walter + Bai AG, Löhningen, Switzerland). This test was performed on three samples with the same composition, and the average value of the compressive strength was calculated.

Thermal analyses were performed with a DIL 402-PC-type (Netzsch, Selb, Germany) horizontal dilatometer with a resolution of 8 μm at a heating rate of 3 °C/min.

## 3. Results and Discussions

### 3.1. Phase Composition and Microstructure of Studied Ceramics

**Figure 3:**
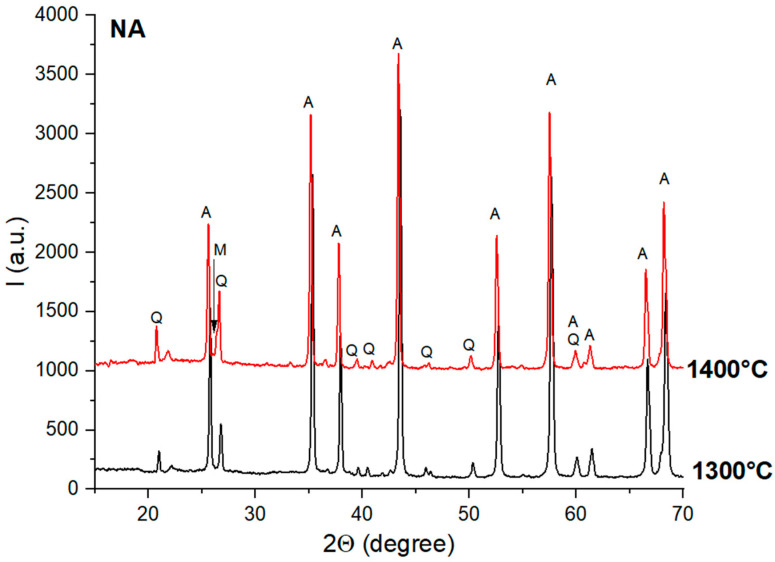
XRD patterns for NA ceramics thermally treated at 1300 °C and 1400 °C; Q—quartz; A—alumina; M—mullite.

The XRD patterns of the NA ceramic samples thermally treated at 1300 °C and 1400 °C are presented in Figure 3.

From Figure 3, one can observe the high crystallinity of the phases present in these samples, i.e., quartz (PDF83-0541) and alumina (PDF-75-0783). The mullite (3Al_2_O_3_·2SiO_2_)—PDF-79-1457—was assessed only for the sample treated at 1400 °C in terms of its specific peaks from 26° and 26.1°, which had a low intensity.

**Figure 4:**
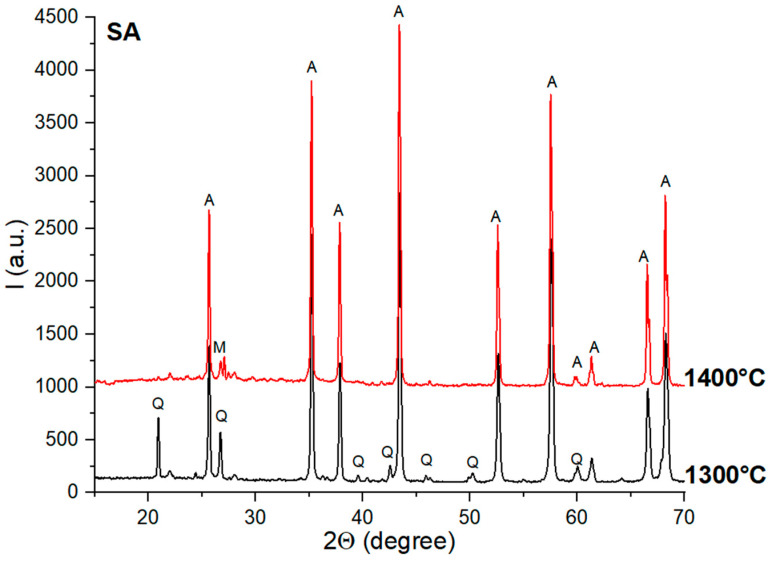
XRD patterns for SA ceramics thermally treated at 1300 °C and 1400 °C; Q—quartz; A—alumina; M—mullite.

For the SA ceramics (Figure 4), one can also observe the high crystallinity of quartz and alumina. The mullite was assessed with a certain degree of incertitude and only for the specimens thermally treated at 1400 °C.

**Figure 5:**
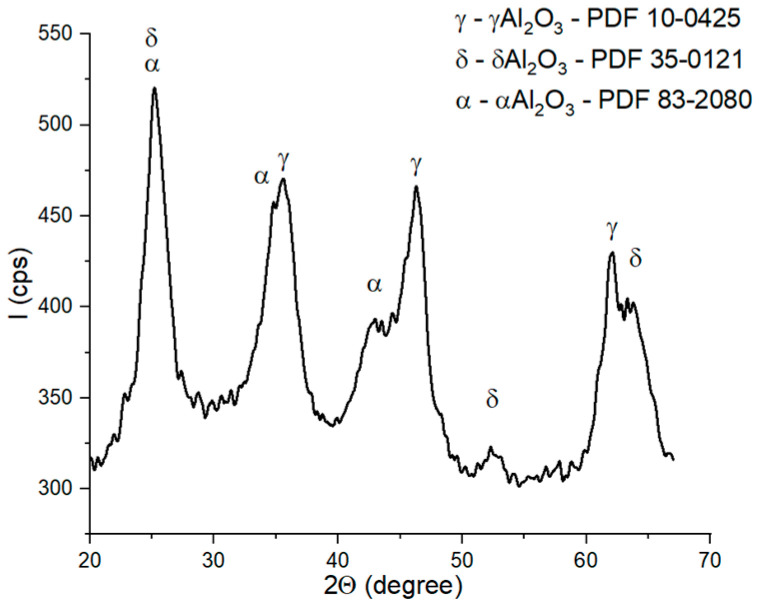
X-ray diffraction patterns of the gel formed by the reaction of aluminum chloride and ammonia solutions after calcination at 350 °C for 2 h.

The main reason why mullite was not formed as the main phase in these ceramics can be explained by the formation of α-Al_2_O_3_ (along with γ- and δ-alumina) during the thermal treatment applied to the aluminum hydroxide precipitate, which formed at the surface of the N and S particles (see Figure 5). The formation of α-Al_2_O_3_, a compound with a low reactivity, after the preliminary thermal treatment at 350 °C hindered its reaction with quartz, and thus the formation of mullite was suppressed.

**Figure 6:**
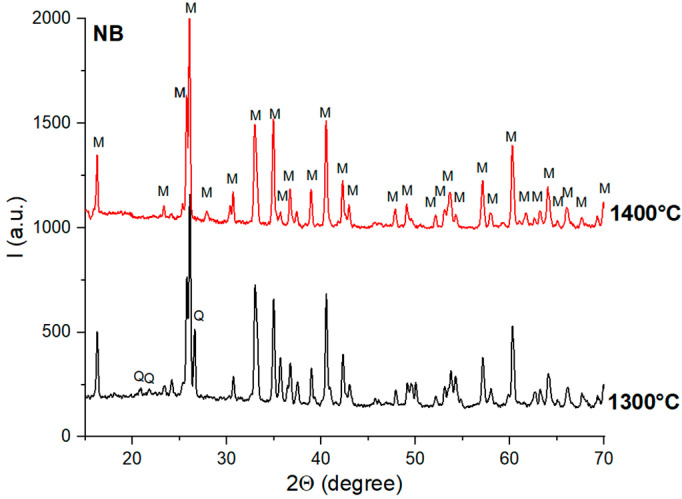
XRD patterns of NB ceramics obtained by thermal treatment at 1300 °C and 1400 °C; Q—quartz; M—mullite.

**Figure 7:**
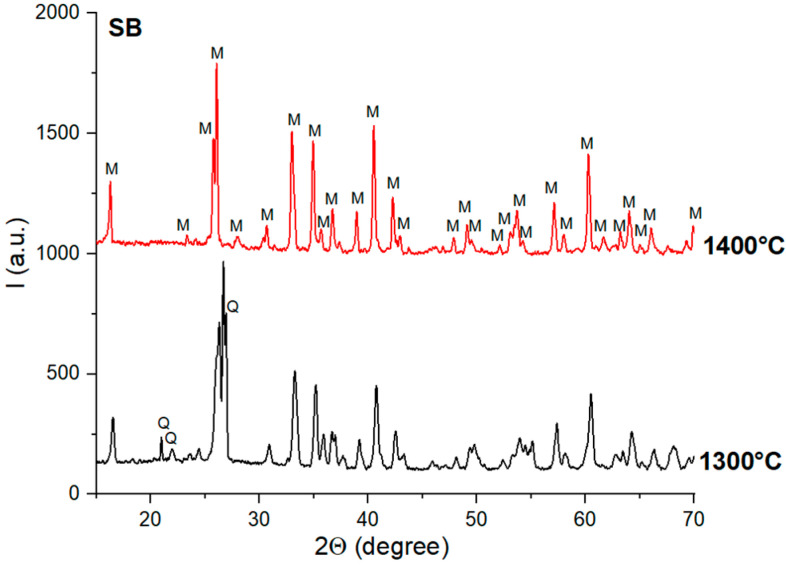
XRD patterns of SB ceramics obtained by thermal treatment at 1300 °C and 1400 °C; Q—quartz; M—mullite.

When the alumina source was bauxite, mullite was observed, along with quartz, on the XRD patterns of the NB and SB ceramics thermally treated at 1300 °C (Figure 6 and Figure 7). The increase in the sintering temperature to 1400 °C determined the formation of the mullite as a single phase.

**Figure 8:**
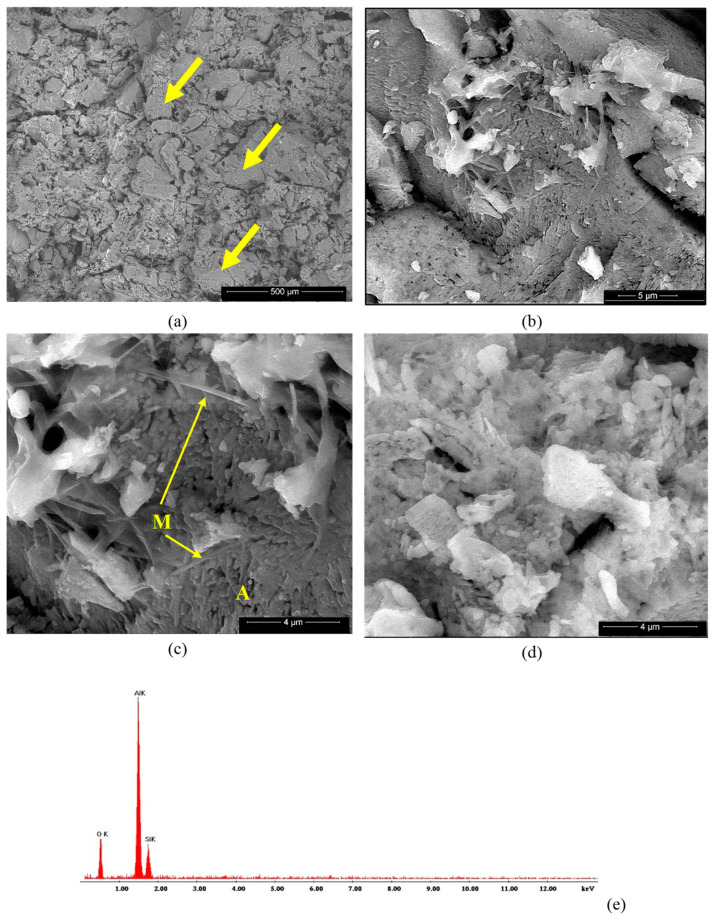
BSE images of NA ceramic obtained by thermal treatment at 1400 °C (**a**–**d**) and EDX spectrum (**e**). Magnifications: (**a**) 200×; (**b**) 10,000×; (**c**) 20,000×; (**d**) 20,000×.

**Figure 9:**
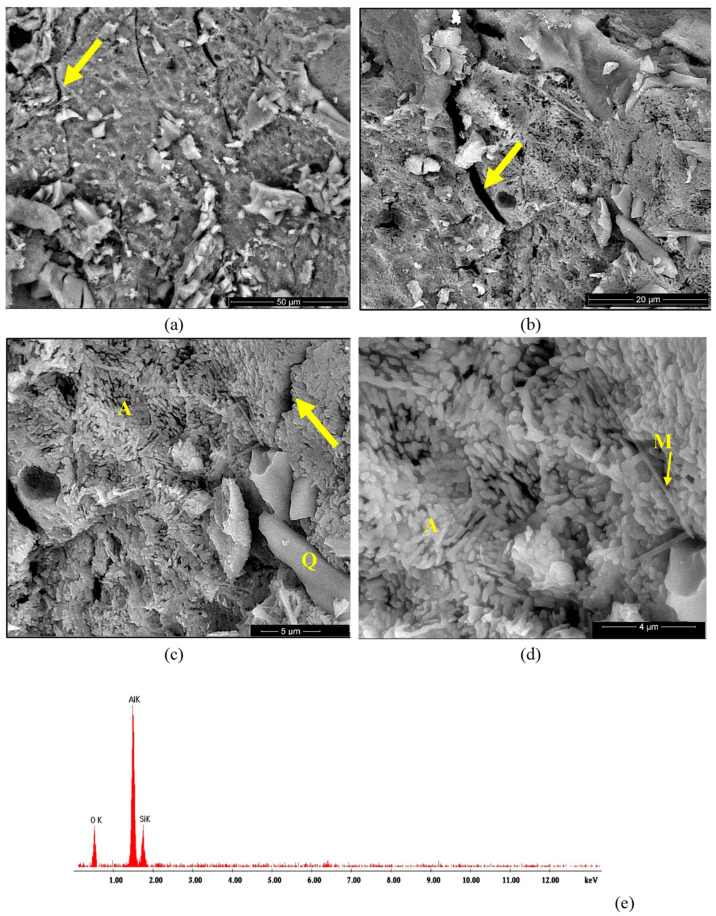
BSE images of SA ceramic obtained by thermal treatment at 1400 °C (**a**–**d**) and EDX spectrum (**e**). Magnifications: (**a**) 2000×; (**b**) 5000×; (**c**) 10,000×; (**d**) 20,000×.

**Figure 10:**
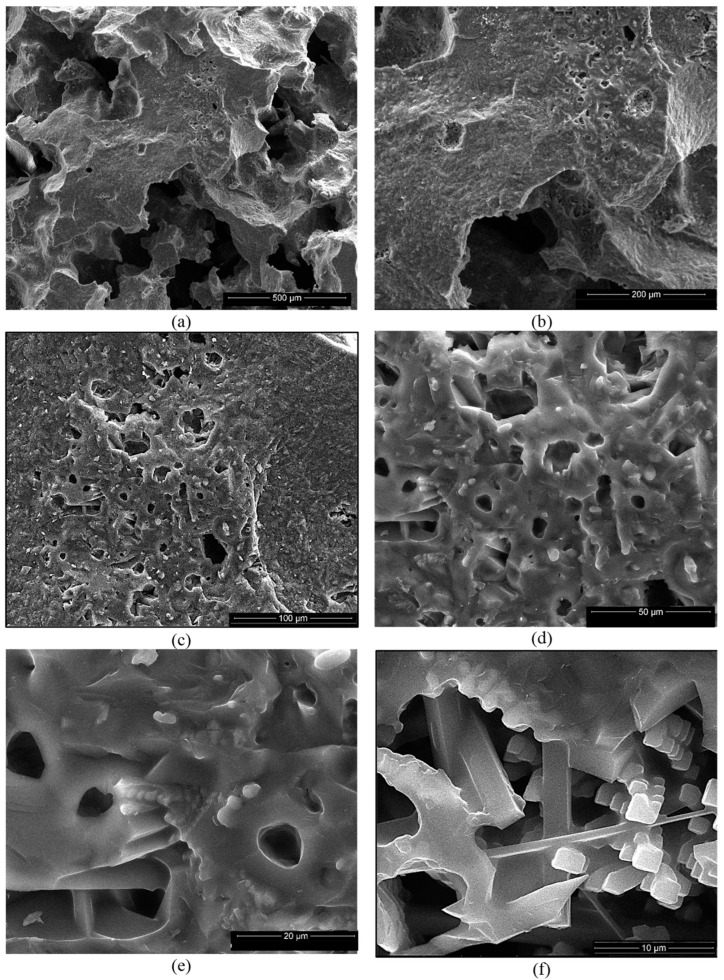

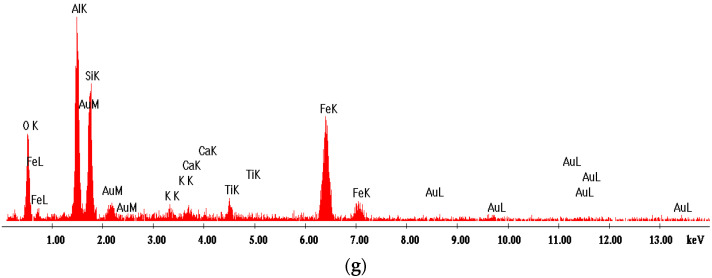
SEM images of SB ceramic obtained by thermal treatment at 1400 °C (**a**–**f**) and EDX spectrum (**g**). Magnifications: (**a**) 200×; (**b**) 500×; (**c**) 1000×; (**d**) 2000×; (**e**) 5000×; (**f**) 10,000×.

**Figure 11:**
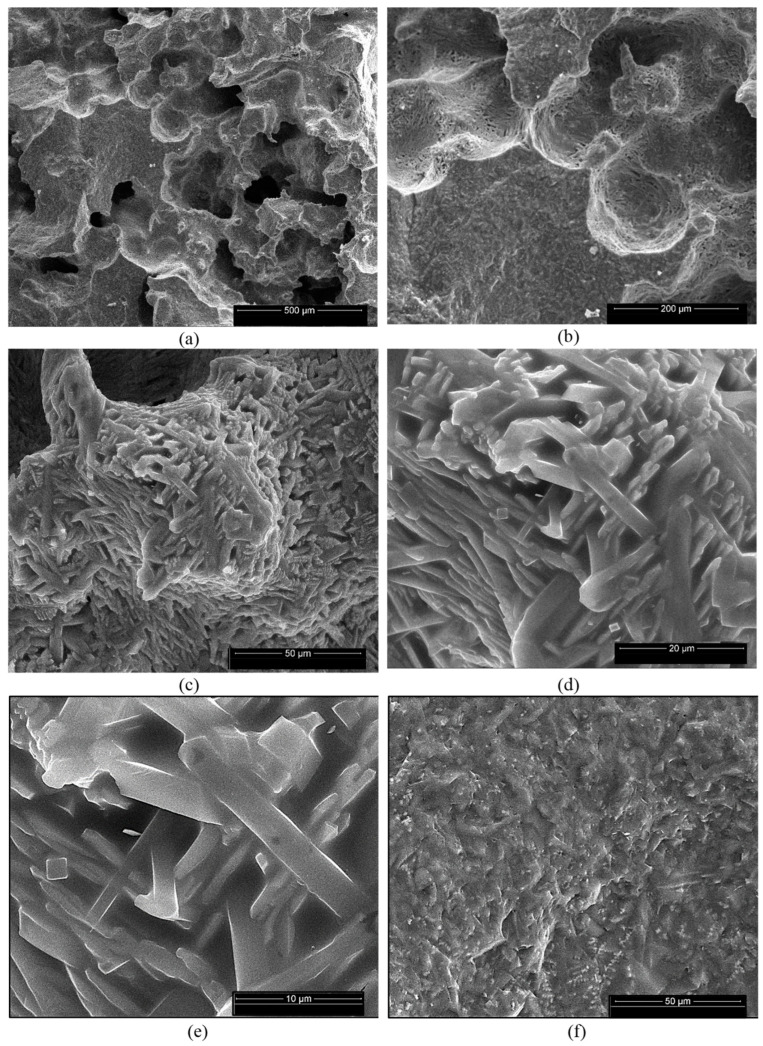

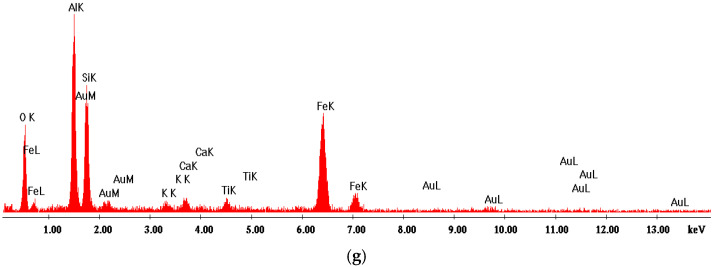
SEM images of NB ceramic obtained by thermal treatment at 1400 °C (**a**–**f**) and EDX spectrum (**g**). Magnifications: (**a**) 200×; (**b**) 500×; (**c**) 2000×; (**d**) 5000×; (**e**) 10,000×; (**f**) 2000×.

The microstructure of the studied ceramics was assessed by SEM, BSE and EDX analyses, as shown in Figure 8, Figure 9, Figure 10 and Figure 11. On the BSE image presented in Figure 8a, one can observe the waste sand N particles (with sharp corners and edges—see arrows) embedded in a porous matrix. At higher magnifications (Figure 8c,d), one can observe the presence of two phases, i.e., small polyhedron crystals (see A in Figure 8c) and needle-like crystals (in a much lower amount), which could be associated with mullite [2]. The EDX spectrum presented in Figure 8e shows the presence of silicium and aluminum along with oxygen in this specimen.

The BSE images of the SA sample (Figure 9a–d) also showed the presence of elongated sand particles (Q) of various sizes embedded in a matrix formed predominantly by small alumina polyhedron crystals (A) along with few mullite crystals (M) with a specific needle-like aspect, as shown in Figure 9d. At a higher magnification (Figure 9c,d), one can notice the independent development of crystalline alumina grains (A) on the surface of the sand grains (Q). This microstructure correlates with the results obtained from the XRD analysis and proves that the alumina (which crystallized very quickly on the surface of the sand grains during the first thermal treatment at 350 °C) had a reduced reactivity and did not quantitatively react with the silica (quartz). Moreover, one can notice multiple cracks (see arrows in Figure 9a–c), which were a result of the individual crystallization of this phase (alumina), which induced stress in the ceramic body. Based on the assessed microstructure, one can consider the SA and NA as composite materials.

For the ceramics obtained using mixtures of bauxite and waste sands S or N, one can notice the formation of a homogeneous porous phase (Figure 10a,b and Figure 11a,b). At a higher magnification, one can observe the formation of the mullite crystals with their specific elongated habitus (Figure 10e,f and Figure 11c–e). One can also notice the presence of a solidified melt (Figure 10d,e and Figure 11f).

The EDX spectrum of these specimens (Figure 10g and Figure 11g) showed the presence of Al, Si and O as the main elements as well as the presence of iron, one of the main components of bauxite.

### 3.2. Ceramic Properties

**Figure 12:**
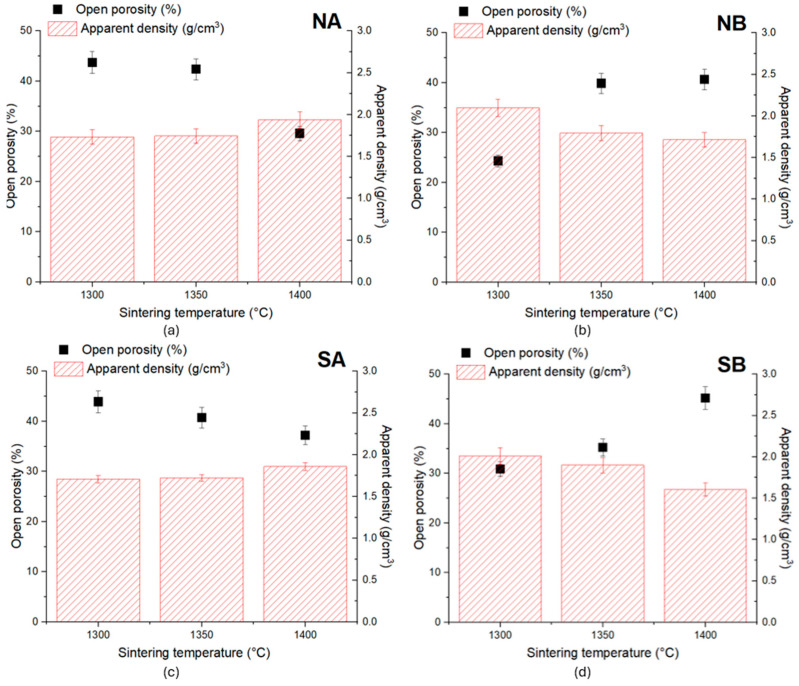
Open porosity and apparent density of ceramic samples sintered at various temperatures: (**a**) NA; (**b**) NB; (**c**) SA; (**d**) SB.

The main properties specific to ceramic materials, i.e., an open porosity and apparent density, are presented in Figure 12.

For the NA ceramic, the reduction in the open porosity was corelated with the increase in the sintering temperature and was more important when the thermal treatment was performed at 1400 °C, i.e., there was a decrease in the open porosity of about 14.14% relative to that recorded for the specimens thermally treated at 1300 °C. One can also notice an increase in the apparent density of 11.87% when the sintering temperature increased from 1300 °C to 1400 °C, while between 1300 °C and 1350 °C, the difference recorded was not significant (0.72%). A similar behavior was assessed for the SA ceramic, i.e., a lower value of the open porosity (and a higher increase in the apparent density) was achieved when the ceramic samples were thermally treated at 1400 °C. This decrease in the porosity could be correlated with the nucleation and growth of alumina crystals with the increase in the sintering temperature (see also the SEM images—Figure 8 and Figure 9).

For the NB ceramic, the highest value of the apparent density (2.1 g/cm^3^) was recorded for the samples thermally treated at 1300 °C. Also, for these samples, the lowest open porosity was observed, i.e., 24.3%. For these ceramics, the increase in the sintering temperature determined a decrease in the apparent density of 18% when the temperature increased from 1300 °C to 1400 °C. At the same time, the open porosity increased by 15.56%, when the sintering temperature increased from 1300 °C to 1350 °C. The further increase in the sintering temperature up to 1400 °C did not have a major influence on the open porosity.

A similar variation in these properties was recorded for the SB ceramics. By increasing the sintering temperature, the apparent density decreased (by 5.5% for the ceramics sintered at 1350 °C and 20.4% for those sintered at 1400 °C). The open porosity increased with the increase in the sintering temperature, with the highest porosity being recorded for the specimens sintered at 1400 °C (an increase of 14.35% relative to that observed for the ceramics thermally treated at 1300 °C).

Opposite to the SA and NA ceramics, the increase in the sintering temperature for the samples containing bauxite as a precursor determines a decrease in the apparent density and an increase in the open porosity. This could be explained by the nucleation and growth of interconnected elongated mullite crystals, contributing to the increase in intragranular porosity (see also Figure 10 and Figure 11).

Thus, the choice of sintering temperature is influenced by the desired properties of the final product.

**Figure 13:**
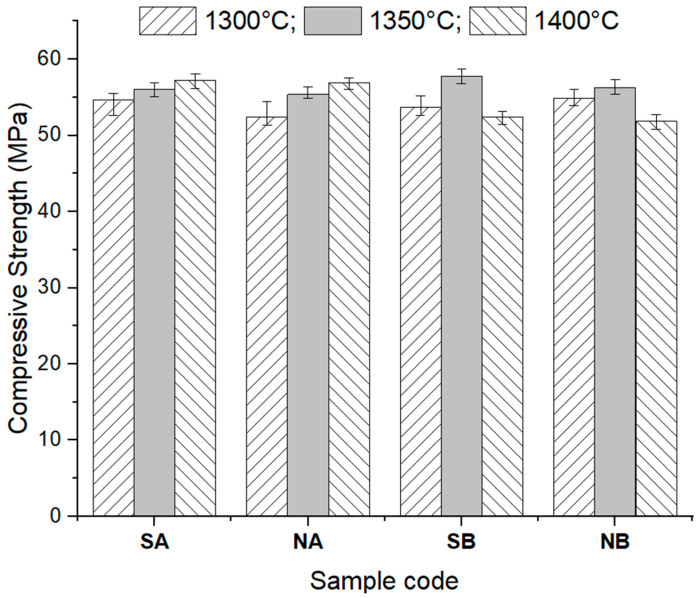
Compressive strength of the studied ceramics sintered at different temperatures.

The compressive strength values, acquired by applying a uniaxial compressive load, of the studied ceramics are presented in Figure 13.

For the NA and SA ceramics, the increase in the sintering temperature determined a small increase in the compressive strengths (8.54% for NA and 4.79% for SA sintered at 1400 °C relative to the values of the same ceramics sintered at 1300 °C), which is explained by the growth of the alumina crystal sizes and the corresponding decrease in the porosity.

For the NB and SB ceramics, the values of the compressive strengths depended on the sintering temperature. The maximum compressive strength value was recorded for the ceramics thermally treated at 1350 °C, followed by a decrease when the temperature increased to 1400 °C. The compressive strength decrease can be explained by the increase in the open porosity (see Figure 12b,d) due to the formation of a higher amount of interconnected elongated mullite crystals.

**Figure 14:**
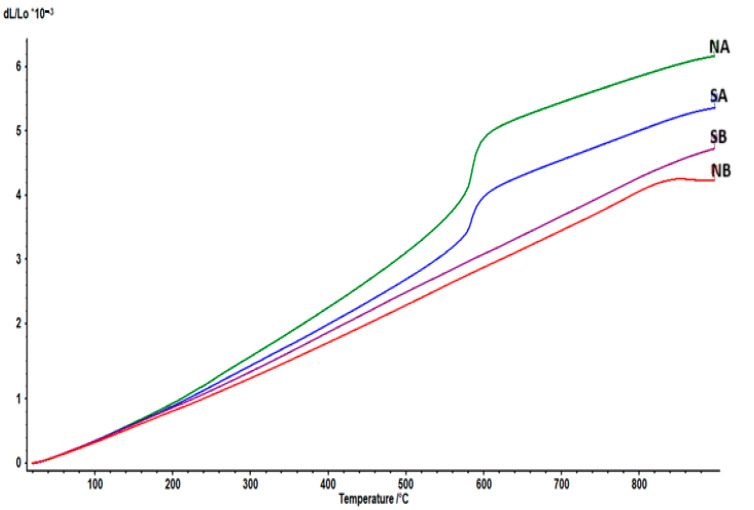
The thermal expansion of the ceramics: NA; NB; SA; SB, sintered at 1350 °C.

The dilatometric curves of the studied ceramics (sintered at 1350 °C) are presented in Figure 14.

The coefficients of thermal expansion (CTE) are presented in Table 2.

For the NA and SA samples, the inflexion at around 575–580 °C could be related to the α–β phase transition in quartz, the main component of silica sand, found in a greater amount in the prepared compositions; this transition is accompanied by relatively large anisotropic changes in cell volume and elastic stiffness properties. Although the transition involves a relatively minor rotation of the silica tetrahedra with respect to one another, it is characterized by a volume change, with the β phase having a larger volume, which leads to higher CTE’s [33].

On the other hand, samples NB and SB had slightly lower CTE’s due to the presence of mullite as the main phase, and they had no phase transition in the 20–900 °C range, thus making them candidates for applications where stability at high temperatures is sought. The softening that occurred at around 840 °C for the NB ceramic can be attributed to the glass-like phase (also observed in the SEM images), which, altogether, had, according to Table 1, larger quantities of silica, alumina, lime, soda and potash than the SB ceramic.

## 4. Conclusions

- Two types of waste sand (N and S) with a high content of impurities were used as precursors for the synthesis of mullite ceramics. Two different synthesis routes were studied: i) solid-state reactions at various temperatures (1300 °C, 1350 °C and 1400 °C) of mixtures of waste sands (S or N) and bauxite (as an alumina precursor) and ii) the precipitation of Al(OH)_3_ on the surface of the sand particles (S or N) followed by a first thermal treatment at 350 °C for alumina formation and then sintering at 1300 °C, 1350 °C and 1400 °C.
- The synthesis method and type of alumina precursors played a fundamental role in the composition of the resulting ceramics; thus, for the ceramics obtained using waste sand (N or S) and bauxite as raw materials, mullite was obtained as the main mineralogic phase after thermal treatment at 1400 °C for 2 h. On the other hand, when alumina (mainly α) was precipitated on the surface of the sand particles, the reaction rate between the alumina and quartz at sintering temperatures between 1300 °C and 1400 °C was low. The BSE images of these specimens showed the formation of a matrix of alumina crystals, in which quartz particles were embedded.
- The values of the apparent density and open porosity of the studied ceramics were influenced by the nature of the formed compounds as well as the sintering temperature. For the ceramics with an alumina content (NA and SA), the increase in the sintering temperature determined an increase in the density and a decrease in the open porosity due to the increase in the sizes of the alumina crystals; for the ceramics with a mullite content (NB and SB), the increase in the sintering temperature had the opposite effect on the open porosity due to the increase in the sizes of the elongated interconnected crystals and, consequently, the increase of the intergranular porosity.
- The compressive strengths of the NA and SA ceramics increased with the increase in the sintering temperature, mainly due to the reduction in the open porosity, in correlation with the microstructure of the ceramics (increase in alumina crystalline grains size); on the other hand, for the ceramics with a mullite content, the increase in the sintering temperature determines a decrease in the compressive strengths due to the increase in the intergranular porosity (due to the specific microstructure—interconnected elongated mullite crystals).
- Due to the specific composition and microstructure of the studied materials, the thermal expansion coefficients (CTEs) of the NA and SA ceramic composites were higher compared to those of the ceramics in which mullite was the main phase, i.e., NB and SB.
- The results presented in this article highlight the importance of the synthesis route correlated with the nature of the precursors, the type and amount of impurities and the sintering temperature.

## Figures and Tables

**Table 1:** The oxide composition of the raw materials.

Waste	L.O.I *. (%)	Oxide Composition (%)								
		SiO_2_	Al_2_O_3_	CaO	Fe_2_O_3_	MgO	SO_3_	Na_2_O	K_2_O	Other Oxides (TiO_2_, V_2_O_5_, MnO, P_2_O_5_, a.o. **)
Waste S	4.26	83.66	2.19	0.67	5.36	0.23	0.10	0.12	0.46	2.95
Waste N	1.29	90.08	3.05	3.20	0.77	0.20	0.07	0.52	0.82	-
Bauxite (B)	11.88	8.13	50.47	1.01	24.39	0.44	-	0.09	0.08	3.51

* L.O.I—loss of ignition; ** a.o.—and other oxides.

**Table 2:** The coefficients of thermal expansion of the studied ceramics.

Sample	Thermal Expansion Coefficient $a_{20}^{900}$ [10^6^ K^−1^]
NA	7.2195
NB	5.0163
SA	6.3086
SB	5.5827

## Data Availability

The original contributions presented in this study are included in the article. Further inquiries can be directed to the corresponding author.

## References

[1] Romero M., Padilla I., Contreras M., López-Delgado A. (2021). Mullite-Based Ceramics from Mining Waste: A Review. Minerals.

[2] Choo T., Mohd Salleh M., Kok K., Matori K. (2019). A Review on Synthesis of Mullite Ceramics from Industrial Wastes. Recycling.

[3] Dong Y., Diwu J., Feng J., Feng X., Liu X., Meng G. (2008). Phase evolution and sintering characteristics of porous mullite ceramics produced from the fly ash–Al(OH)_3_ coating powders. J. Alloys Compd..

[4] Roy R., Das D., Rout P. (2022). A Review of Advanced Mullite Ceramics. Eng. Sci..

[5] Schneider H., Schreuer J., Hildmann B. (2008). Structure and properties of mullite—A review. J. Eur. Ceram. Soc..

[6] Chen C., Lan G., Tuan W. (2000). Preparation of mullite by the reaction sintering of kaolinite and alumina. J. Eur. Ceram. Soc..

[7] Bella M.L., Hamidouche M., Gremillard L. (2021). Preparation of mullite-alumina composite by reaction sintering between Algerian kaolin and amorphous aluminum hydroxide. Ceram. Int..

[8] Song K. (1998). Preparation of Mullite Fibers by the Sol-Gel Method. J. Sol-Gel Sci. Technol..

[9] Won C.W., Siffert B. (1998). Preparation by sol-gel method of SiO_2_ and mullite (3Al_2_O_3_·2SiO_2_) powders and study of their surface characteristics by inverse gas chromatography and zetametry. Colloids Surf. A Physicochem. Eng. Asp..

[10] Ilić S., Babić B., Bjelajac A., Stoimenov N., Kljajević L., Pošarac–Marković M., Matović B. (2020). Structural and morphological characterization of iron-doped sol-gel derived mullite powders. Ceram. Int..

[11] Sotirchos S.V., Nitodas S.F. (2002). Factors influencing the preparation of mullite coatings from metal chloride mixtures in CO_2_ and H_2_. J. Cryst. Growth.

[12] Xu J., Erickson D., Roy S., Sarin V. (2013). Protective CVD Mullite Coatings on Single-Crystal Silicon Substrates. JOM.

[13] Dabbs D., Yao N., Aksay I. (1999). Nanocomposite Mullite/Mullite Powders by Spray Pyrolysis. J. Nanopart. Res..

[14] Janackovic D., Jokanovic V., Kostic-Gvozdenovic L., Uskokovic D. (1998). Synthesis of mullite nanostructured spherical powder by ultrasonic spray pyrolysis. Nanostruct. Mater..

[15] European Commission Circular Economy Action Plan. https://environment.ec.europa.eu/strategy/circular-economy-action-plan_en.

[16] Lupu O., Ardelean M., Socalici A., Ardelean E. (2021). Research regarding the capitalization of waste resulted from the steel industry. Univ. Politeh. Buchar. Sci. Bull. Ser. B Chem. Mater. Sci..

[17] Chen L., Yang M., Chen Z., Xie Z., Huang L., Osman A., Farghali M., Sandanayake M., Liu E., Ahn Y.H. (2024). Conversion of waste into sustainable construction materials: A review of recent developments and prospects. Mater. Today Sustain..

[18] Dima-Vadauva C., Badanoiu A., Nicoara A. (2020). Properties of cement-based composites with chopped electrical cables and polyurethane wastes. Univ. Politeh. Buchar. Sci. Bull. Ser. B.

[19] Hossain S., Pyare R., Roy P.K. (2020). Synthesis of in-situ mullite foam using waste rice husk ash derived sol by slip-casting route. Ceram. Int..

[20] Alves H., Silva J., Campos L., Torres S., Dutra R., Macedo D. (2016). Preparation of mullite based ceramics from clay–kaolin waste mixtures. Ceram. Int..

[21] Khalil N., Algamal Y. (2020). Recycling of ceramic wastes for the production of high performance mullite refractories. Silicon.

[22] Liu R., Xiang D. (2012). Recycling photovoltaic silicon waste for fabricating porous mullite ceramics by low-temperature reaction sintering. J. Eur. Ceram. Soc..

[23] López-Cuevas J., Interial-Orejón E., Gutiérrez-Chavarría C., Rendón-Ángeles J. (2017). Synthesis and Characterization of Cordierite, Mullite and Cordierite-Mullite Ceramic Materials using Coal Fly Ash as Raw Material. MRS Adv..

[24] Koshy P., Ho N., Zhong V., Schreck L., Koszo S., Severin E., Sorrell C. (2021). Fly Ash Utilisation in Mullite Fabrication: Development of Novel Percolated Mullite. Minerals.

[25] Ribeiro M., Labrincha J. (2008). Properties of sintered mullite and cordierite pressed bodies manufactured using Al-rich anodising sludge. Ceram. Int..

[26] Wang W., Shi Z., Wang Z., Wang S. (2018). Phase transformation and properties of high-quality mullite ceramics synthesized using desert drift sands as raw materials. Mater. Lett..

[27] Tripathi H.S., Banerjee G. (1998). Synthesis and mechanical properties of mullite from beach sand sillimanite: Effect of TiO_2_. J. Eur. Ceram. Soc..

[28] Tripathi H.S., Banerjee G. (1998). Synthesis and Mechanical Properties of Mullite Developed from Beach Sand Sillimanite: Effect of Fe_2_O_3_. Trans. Indian Ceram. Soc..

[29] Sánchez-Soto P., Eliche-Quesada D., Martínez-Martínez S., Garzón-Garzón E., Pérez-Villarejo L., Rincón J. (2018). The effect of vitreous phase on mullite and mullite-based ceramic composites from kaolin wastes as by-products of mining, sericite clays and kaolinite. Mater. Lett..

[30] Cui Z., Hao T., Yao S., Hu H. (2023). Preparation of porous mullite ceramic supports from high alumina fly ash. J. Mater. Cycles Waste Manag..

[31] Stoleriu S., Andronescu E., Carabat A., Vasile B.S. (2011). Influence of preparation conditions on nanaometric characteristics of zirconia and alumina powders. Rom. J. Mater..

[32] (1995). Ceramic Tiles—Part 3. Determination of Water Absorption, Apparent Porosity, Apparent Relative Density and Bulk Density.

[33] Johnson S., Song W., Cook A., Vel S., Gerbi C. (2021). Quartz A↔Β Phase Transition: Does it drive damage and reaction in continental crust?. Earth Planet. Sci. Lett..

